# Clinical updates of JAK inhibitors in cutaneous granulomatous diseases

**DOI:** 10.3389/fimmu.2025.1698816

**Published:** 2025-12-12

**Authors:** Jiaxu Gu, Xinglin He, Bingcheng Lu, Jieyi Wang, Kexin Chen, Qiaofen Wang, Xingling Jian, Cong Huang, Bo Yu

**Affiliations:** 1 Department of Dermatology, Peking University Shenzhen Hospital, Shenzhen, China; 2 Shenzhen Key Laboratory for Translational Medicine of Dermatology, Shenzhen Peking University - The Hong Kong University of Science and Technology Medical Center, Shenzhen, China; 3 Department of Dermatology, Shenzhen Xinhua Hospital, Shenzhen, China; 4 Department of Rheumatology, Hainan Provincial People’s Hospital, Haikou, China

**Keywords:** JAK inhibitors, cutaneous granulomatous diseases, JAK-STAT pathway, targeted therapy, clinical practice

## Abstract

Cutaneous granulomatous diseases, characterized by persistent granuloma formation, often exhibit chronic and relapsing courses that are challenging to manage with conventional therapies. The Janus kinase (JAK)–signal transducer and activator of transcription (STAT) pathway plays a central role in mediating key cytokines involved in granuloma initiation and maintenance, such as IFN-γ, IL-6, IL-12, and IL-23. JAK inhibitors, by targeting this pathway, offer a promising therapeutic strategy for refractory cases. This review synthesizes current evidence supporting the efficacy of JAK inhibitors—including tofacitinib, ruxolitinib, baricitinib, upadacitinib, and abrocitinib—in conditions such as sarcoidosis, granuloma annulare, granulomatous rosacea, and adverse reactions to cosmetic injectables. Clinical studies and case reports have demonstrated that JAK inhibitors significantly improve lesion outcomes and effectively control symptoms in these conditions, highlighting their potential as targeted treatments. However, further large-scale trials are needed to establish optimal dosing, long-term safety, and predictive biomarkers for personalized therapy.

## 1 Introduction

Cutaneous granulomatous diseases represent a heterogeneous group of disorders unified by the common pathological feature of granuloma formation (1). Their etiologies are diverse, encompassing infections, autoimmune dysfunctions, foreign body reactions, and idiopathic factors (2). These conditions manifest with complex and varied clinical presentations, often following a chronic and relapsing course that significantly impairs patients’ quality of life (3). Conventional therapeutic strategies, including corticosteroids, immunosuppressants (such as methotrexate), and biological agents, often yield suboptimal outcomes for some patients, characterized by inadequate efficacy, high relapse rates, or significant adverse effects (4–6). Consequently, there is a pressing clinical need to explore novel and more targeted treatment approaches.

The Janus kinase (JAK) - signal transducer and activator of transcription (STAT) signaling pathway has emerged as a major research focus in immunology and dermatology in recent years. It plays a pivotal role in the signal transduction of numerous cytokines—such as Interferon-gamma (IFN-γ), IL-2, IL-4, IL-6, IL-12, IL-15, and IL-23—which are key mediators driving granuloma formation and sustaining inflammatory processes (7, 8). JAK inhibitors are a class of small-molecule targeted therapeutics that attenuate the signaling of multiple pro-inflammatory cytokines by inhibiting the JAK-STAT pathway, either orally or topically (9). Mounting evidence from recent case reports, case series, and early-phase clinical trials indicates that JAK inhibitors exhibit promising therapeutic potential in managing refractory cutaneous granulomatous diseases, such as sarcoidosis and granuloma annulare (10–12).

This review provides a clinically oriented appraisal of the potential of JAK inhibitors in this challenging disease spectrum. Rather than presenting a comprehensive mechanistic treatise, the present review aims to synthesize and critically evaluate the current clinical evidence—comprising case reports, series, and early trials—that supports the use of various JAK inhibitors. It will candidly discuss the significant limitations and biases inherent in this low-level evidence base and, most importantly, provide a forward-looking roadmap for future clinical research and trial design, outlining the necessary steps to translate this preliminary promise into robust, evidence-based clinical practice.

## 2 JAK-STAT signaling

The JAK-STAT signaling pathway is a central intracellular transduction mechanism mediating the signals of numerous cytokines, growth hormones, and interferons. Pathway activation is initiated by the binding of extracellular cytokines to their cognate receptors, which induces transphosphorylation and activation of the associated Janus kinases (JAKs), including JAK1, JAK2, JAK3, and TYK2 (13). The activated JAKs then phosphorylate the intracellular domains of the receptors, creating docking sites for STAT proteins (STAT1, STAT2, STAT3, STAT4, STAT5A, STAT5B, and STAT6) (14–16). Upon phosphorylation, STAT proteins form dimers, translocate to the nucleus, and function as transcription factors to regulate the expression of target genes, thereby playing broad roles in immune responses, inflammatory reactions, cell survival, and proliferation (17, 18).

It is noteworthy that the JAK-STAT pathway demonstrates both specificity and redundancy in its function. For instance, IFN-γ signaling primarily involves JAK1 and JAK2 to activate STAT1, whereas IL-6 signaling can engage various combinations such as JAK1, JAK2, and/or TYK2 to ultimately activate STAT3 (19). This characteristic implies that broad inhibition of JAK proteins can simultaneously block the effects of multiple cytokines, providing a therapeutic target for autoimmune diseases (20–24). However, selective inhibition of specific JAK subtypes may yield more targeted therapeutic effects and influence the safety profile of such interventions (25–27).

## 3 Metabolic reprogramming in granulomatous inflammation: the interplay of immune signaling and metabolic dysregulation

Emerging evidence underscores that metabolic reprogramming is a fundamental driver of immune cell function in chronic inflammatory diseases. In cutaneous granulomatous disorders, recent findings have revealed a critical axis in which immune signaling directly orchestrates metabolic shifts to sustain the granulomatous response.

A seminal study by Nakamizo et al. has identified the transcription factor c-Maf as a key regulator of macrophage metabolism within granulomas (28). Their work demonstrated that c-Maf, which is upregulated in granulomatous macrophages, acts as a metabolic switch by suppressing glycolysis and mitochondrial respiration while concurrently enhancing the pentose phosphate pathway (PPP). This specific reprogramming is essential for supporting the unique biosynthetic and redox demands of chronic macrophage activation and directly contributes to granuloma formation. The upregulation of the PPP, a branch of glucose metabolism, is particularly crucial. The PPP yields two key products: ribose-5-phosphate and NADPH. Ribose-5-phosphate serves as a precursor for *de novo* nucleotide synthesis, which is indispensable for the clonal expansion of antigen-specific T cells and the localized proliferation of macrophages within the granuloma structure (29, 30). Concurrently, NADPH plays a dual role: it maintains cellular redox balance by regenerating glutathione, thereby protecting macrophages from oxidative stress-induced apoptosis in the inflammatory microenvironment, and it serves as an essential cofactor for NADPH oxidase (NOX2) to generate reactive oxygen species (ROS) for microbial killing (31, 32). The c-Maf-driven metabolic shift ensures a steady supply of these metabolites, thereby maintaining the viability and pro-inflammatory function of granulomatous macrophages (33, 34).

This metabolic reprogramming is intricately linked to and potentiated by canonical pro-inflammatory signaling. Key cytokines implicated in granuloma pathogenesis, such as IFN-γ and IL-6, signal through their respective JAK-STAT axes (JAK1/JAK2-STAT1 and JAK1/JAK2/TYK2-STAT3) (35, 36). These pathways can further amplify the PPP, creating a feed-forward loop that sustains inflammation. For example, STAT activation can regulate the expression of rate-limiting enzymes in the PPP, such as glucose-6-phosphate dehydrogenase (G6PD) (37, 38). The resulting metabolic state—characterized by PPP dependency—is a hallmark of the classically activated (M1) macrophage phenotype and is critical for sustaining the high ROS output and biosynthetic capacity required for granuloma maintenance (37, 39, 40).

Overall, the pathogenesis of granulomatous diseases is no longer viewed solely through an immunological lens but is now understood as an integrated network of immune and metabolic circuits. The JAK-STAT pathway and transcription factors like c-Maf converge to rewire core metabolic pathways, such as the PPP, to fuel the persistent inflammatory state.

### 3.1 The relationship between JAK-STAT signaling and skin granuloma formation

Granuloma formation is a complex immune response to persistent stimuli such as infection, foreign bodies, or autoantigens, characterized by the organized aggregation of macrophages and other immune cells (1). In this process, the JAK-STAT signaling pathway plays a central role in the initiation, maintenance, and progression of the disease by mediating the signal transduction of multiple key cytokines (**Table 1**).

**Table 1:** Key Cytokine-JAK-STAT axes and their pathogenic roles in cutaneous granuloma formation.

Cytokine	Primary cellular source	Receptor complex	JAK(s) involved	STAT(s) activated	Downstream target genes / key molecules	Specific roles in granuloma pathogenesis	Associated granulomatous diseases
IFN-γ	Th1 cells, NK cells, CD8+ T cells	IFNGR1 / IFNGR2	JAK1, JAK2	STAT1 (homodimer)	*IRF1, CXCL9, CXCL10, MHC class II, NOS2*	• M1 Macrophage Polarization: Induces classical activation.	Sarcoidosis, Granuloma Annulare, Infectious Granulomas (e.g., Tuberculosis)
						• Antigen Presentation: Upregulates MHC-II, enhancing T cell recognition.	
						• Lymphocyte Recruitment: Drives CXCL9/10 production, recruiting CXCR3+ T cells.	
						• Microbial Clearance: Promotes reactive oxygen/nitrogen species production.	
IL-6	Macrophages, Dendritic cells, Fibroblasts	IL-6R / gp130	JAK1, JAK2, TYK2	STAT3	*Bcl-2, Bcl-xL, Survivin, MCP-1, CRP*	• Cell Survival & Anti-apoptosis: Extends lifespan of macrophages and inflammatory cells.	Sarcoidosis, Granulomatous Rosacea, Rheumatoid Nodules
						• Acute Phase Response: Amplifies local and systemic inflammation.	
						• T Cell Activation: Co-stimulates T cell proliferation and differentiation.	
IL-12	Activated Macrophages, Dendritic cells	IL-12Rβ1 / IL-12Rβ2	TYK2, JAK2	STAT4	*IFN-γ, TBX21 (T-bet)*	• Th1 Cell Differentiation: Master regulator of Th1 lineage commitment.	Sarcoidosis, Granuloma Annulare
						• IFN-γ Amplification: Drives IFN-γ production from T and NK cells, reinforcing the Th1 circuit.	
IL-23	Activated Macrophages, Dendritic cells	IL-23R / IL-12Rβ1	TYK2, JAK2	STAT3 (also STAT4, STAT5)	*RORγt, IL-17A, IL-17F, IL-22*	• Th17 Cell Expansion & Stability: Critical for maintenance of Th17 population.	Granulomatous Rosacea, Foreign Body Granulomas, Sarcoidosis (subset)
						• Neutrophil Recruitment: Via induction of IL-17, promoting neutrophilic inflammation.	
						• Barrier Dysfunction & Tissue Remodeling: Via IL-22.	
IL-4 / IL-13	Th2 cells, Eosinophils, Basophils	Type II Receptor (IL-4Rα / IL-13Rα1 or γc)	JAK1, JAK3, TYK2	STAT6	*ARG1, CCL17, CCL22, CD206, FIZZ1*	• M2 Macrophage Polarization: Induces alternative activation.	Chronic Granulomatous Diseases with fibrosis, Helminth Infections
						• Fibrosis Promotion: Stimulates fibroblast proliferation and collagen production.	
						• Resolution & Repair: Modulates inflammation but can contribute to fibrotic sequelae.	
TGF-β1	Macrophages, Tregs, Fibroblasts, Platelets	TGFβR-I / TGFβR-II	(Smad-dependent pathway)	(Smads 2/3)	*COL1A1, COL3A1, ACTA2 (α-SMA), PAI-1*	• Myofibroblast Differentiation: Key driver of fibrosis.	All chronic granulomatous diseases (Late-stage fibrosis)
						• Extracellular Matrix Deposition: Stimulates collagen and fibronectin production.	
						• Immune Regulation: Can suppress T cell function but also promotes Th17 differentiation in context of IL-6.	

The process is initiated by the release of key cytokines. IFN-γ, by activating the JAK1/JAK2–STAT1 signaling axis in macrophages, drives their polarization toward the pro-inflammatory M1 phenotype (41). This enhances MHC class II expression and antigen presentation capacity, while also upregulating chemokines such as CXCL9 and CXCL10, which recruit T cells to the inflammatory site (42–44). Simultaneously, IL-6 signals through the JAK1/JAK2/TYK2–STAT3 pathway, promoting the expression of anti-apoptotic genes like Bcl-2, thereby extending macrophage survival and sustaining the local inflammatory state (45, 46).

These early innate immune responses pave the way for the activation of adaptive immunity. Recruited CD4^+^ T cells differentiate into effector subsets under the influence of specific cytokines (47). IL-12, signaling via the TYK2/JAK2–STAT4 pathway, directs Th1 cell differentiation, while IL-23, through the TYK2/JAK2–STAT3 pathway, promotes the generation and maintenance of Th17 cells (48, 49). Differentiated Th1 cells secrete large amounts of IFN-γ, establishing a positive feedback loop with macrophages that exacerbates inflammation (50). Th17 cells, in turn, produce IL-17 and IL-22, further amplifying the inflammatory response and recruiting neutrophils (51).

The interactions between macrophages and T cells, mediated by JAK-STAT pathways, collectively establish a self-amplifying inflammatory microenvironment essential for the formation and maintenance of granulomatous structures. In the chronic phase, persistent antigenic stimulation leads to repeated activation of STAT1 and STAT3 (52). This not only sustains elevated expression of pro-inflammatory mediators and chemokines but also upregulates the transcription of pro-fibrotic genes such as TGF-β1 and collagen, ultimately resulting in tissue damage and fibrosis (53, 54). Similarly, STAT3 activation via IL-6 and other gp130-family cytokines promotes the expression of anti-apoptotic genes such as Bcl-2 and survivin, thereby extending the lifespan of macrophages and maintaining granuloma architecture (55, 56). Furthermore, negative regulatory mechanisms of the JAK-STAT pathway, such as suppressors of cytokine signaling (SOCS) proteins, are often dysregulated in chronic granulomatous diseases, preventing the proper termination of inflammatory signaling (57, 58).

## 4 JAK inhibitors

JAK inhibitors are a class of small-molecule therapeutics designed to selectively inhibit one or more members of the Janus kinase family (JAK1, JAK2, JAK3, TYK2), thereby modulating the JAK-STAT signaling pathway. Therapeutic targeting of JAK-STAT components with inhibitors (e.g., tofacitinib, ruxolitinib) can disrupt this vicious cycle by blocking downstream phosphorylation and nuclear translocation of STATs, thereby reducing cytokine production and immune cell activation, as evidenced in preclinical and clinical studies of sarcoidosis and granuloma annulare. By blocking the phosphorylation and activation of STAT proteins, these agents reduce the downstream effects of multiple cytokines implicated in inflammation, immune activation, and granuloma formation. Their oral and topical formulations offer flexible administration routes, making them suitable for both systemic and localized diseases. Beyond their well-established use in rheumatoid arthritis and myeloproliferative disorders, JAK inhibitors are increasingly applied in dermatology – particularly in conditions driven by dysregulated innate and adaptive immune responses (59–61). Their ability to interfere with key pro-inflammatory cytokines such as IFN-γ, IL-4, IL-6, IL-12, IL-13, and IL-23 underpins their therapeutic potential in a range of inflammatory and autoimmune skin diseases (**Table 2**).

**Table 2:** JAK Inhibitors Used in dermatological practice.

Generic name	Example brand name	Primary target	Approved dermatologic indications	First approved (FDA, unless noted)	Formulation & strength	Typical dermatologic dosage	Notes
Tofacitinib	Xeljanz^®^	JAK1/JAK3	Atopic Dermatitis (AD) Psoriatic Arthritis (PsA)	2012	Oral tablets: 5mg, 10mg	5 mg twice daily	The first JAK inhibitor approved. Has extensive off-label use data in dermatology.
			Off-label: Alopecia areata cutaneous sarcoidosis granuloma annulare				
Ruxolitinib	Opzelura^®^	JAK1/JAK2	Non-segmental vitiligo (age 12+) AD	2021 (Vitiligo), 2022 (AD)	Topical cream: 1.5%	Apply a thin layer to affected areas twice daily	First and only approved topical JAK inhibitor. Systemic formulation (Jakafi^®^) is used off-label.
Baricitinib	Olumiant^®^	JAK1/JAK2	Severe AD in adults	2020 (EU), 2022 (FDA)	Oral tablets: 2mg, 4mg	4 mg once daily (2 mg may be used for dose reduction)	One of the first oral JAK inhibitors approved for AD.
Upadacitinib	Rinvoq^®^	JAK1	Moderate-to-severe AD in adults and adolescents (age 12+), PsA, Alopecia areata(AA)	2019 (RA), 2022 (AD)	Oral tablets: 15mg, 30mg	AD: 15 mg or 30 mg once daily	Shows high efficacy in AD clinical trials. Used off-label for granuloma annulare.
Abrocitinib	Cibinqo^®^	JAK1	Moderate-to-severe AD in adults	2021	Oral tablets: 50mg, 100mg, 200mg	100 mg or 200 mg once daily	Selective JAK1 inhibitor. Case reports of use in granuloma annulare and filler-induced granulomas.
Delgocitinib	Corectim^®^ Ointment	Pan-JAK	AD (Japan)	2020 (Japan PMDA)	Topical ointment: 0.5%	Apply twice daily	

The efficacy of JAK inhibitors has garnered particular interest in the management of granulomatous skin disorders, including sarcoidosis, granuloma annulare, adverse reactions to cosmetic injectables and granulomatous rosacea. These conditions are characterized by the formation of immune cell aggregates and persistent activation of macrophage and T-cell pathways, largely mediated by dysregulated JAK-STAT signaling. Early preclinical and clinical reports suggest that both topical and oral JAK inhibitors can improve granuloma formation and cutaneous inflammation with a favorable safety profile (Refs). As research advances, these targeted agents hold promise as novel therapeutic strategies for refractory granulomatous dermatoses.

## 5 Clinical application of JAK inhibitors in cutaneous granulomatous diseases

### 5.1 Sarcoidosis

Sarcoidosis is a classic non-infectious granulomatous disorder, most commonly manifesting as pulmonary nodules. However, approximately 30% of patients develop cutaneous symptoms, which may present as plaques, nodules, or lupus pernio (62). Sarcoid granulomas are composed of macrophages and T cells that secrete IL-6 and IFN-γ. Within these lesions, STAT1 is primarily activated in macrophages, whereas STAT3 shows activation among lymphocytic aggregates surrounding the granulomas (63). Molecular analyses have demonstrated that oral tofacitinib reduces levels of proinflammatory cytokines in patients with cutaneous sarcoidosis (64). Additionally, there have been reports of successful treatment of cutaneous sarcoidosis, including cases with comorbidities, using ruxolitinib and baricitinib (65, 66). These findings collectively highlight the considerable potential of JAK inhibitors in the management of sarcoidosis.

### 5.2 Granuloma annulare

In granuloma annulare, a Th1-polarized immune response drives aberrant activation of the JAK-STAT pathway, leading to the development and progression of the granulomatous lesions. Activated T cells secrete large amounts of IFN-γ, which binds to receptors on the surface of macrophages and activates associated JAK1 and JAK2 kinases (67). This in turn phosphorylates and activates the transcription factor STAT1. Activated STAT1 translocates into the nucleus, where it initiates the transcription of a series of pro-inflammatory genes (68). This process directly promotes macrophage activation, proliferation, and M1-polarization, and stimulates the secretion of cytokines such as TNF-α (69). These events ultimately lead to collagen degradation and lymphocytic aggregation, resulting in the formation of the characteristic palisaded granulomatous structure. Clinical evidence indicates that the use of JAK inhibitors, such as upadacitinib and abrocitinib, can effectively block this signaling axis and significantly improve GA skin lesions, thereby providing therapeutic confirmation of the central role of the JAK-STAT pathway in the pathogenesis of granuloma annulare (70–72).

### 5.3 Granulomatous rosacea

Rosacea is a chronic inflammatory disorder whose pathogenesis involves the interaction between genetic and environmental factors, dysregulation of the innate immune system, neurovascular modifications, and their interplay with the skin (73). Among the subtypes of rosacea, granulomatous rosacea represents a distinct variant, primarily affecting the perinasal area and characterized histopathologically by typical granulomatous lesions (74). The JAK/STAT signaling pathway plays a significant role in the pathogenesis of granulomatous rosacea. Previous studies have demonstrated that in an LL-37-induced rosacea-like mouse model, topical application of tofacitinib significantly improved rosacea-like phenotypes, reduced CD4^+^ T cell and mast cell infiltration, and suppressed dermal angiogenesis. RT-qPCR analysis revealed decreased mRNA expression levels of STAT1, STAT4, and STAT5a in the lesions after topical tofacitinib treatment (75). In addition to basic research, clinical applications have also confirmed these findings. A 53-year-old woman underwent a skin biopsy, which revealed granulomatous inflammation in the lesion area. Both acid-fast staining and periodic acid–Schiff (PAS) staining yielded negative results. Based on clinical manifestations and laboratory findings, the patient was diagnosed with granulomatous rosacea. After 20 weeks of treatment with the JAK-1 inhibitor abrocitinib, the skin rash and associated burning sensation substantially improved. Subsequent follow-up indicated no adverse reactions or recurrence (76).

### 5.4 Adverse reactions to cosmetic injectables

In the field of modern aesthetic medicine, cosmetic injectables are increasingly chosen by a wide range of beauty seekers due to their simplicity, minimally invasive nature, and relatively affordable cost (77). However, the incidence of adverse reactions has been rising as a result of issues such as substandard materials, improper injection techniques, and inadequate sterilization practices. Among all adverse reactions, chronic and disfiguring nodules (or granulomas) and swelling following cosmetic injections are one of the most common. A study showed that in their statistical analysis of histological examinations of skin lesions following adverse reactions to injections, 87.1% of patients had foreign body granulomas, and 3% had inflammatory granulomas (78). In the context of foreign body granuloma (FBG), JAK inhibitors exert therapeutic effects through multi-level immunomodulatory mechanisms. Their actions primarily include: disrupting T cell-macrophage homeostasis, inhibiting STAT6-dependent multinucleated giant cell (MGC) formation; suppressing pro-fibrotic cytokines such as TGF-β1 and IL-13 at the transcriptional level, while attenuating macrophage polarization and T cell-mediated inflammatory pathways; and downregulating M1 macrophage markers (TNF-α, CXCL10) to block key inflammatory mediators (79–82). Ultimately, by inhibiting the IFN-γ-driven JAK-STAT signaling pathway, they comprehensively intervene in multiple pathophysiological aspects of granulomatous inflammation. The emergence of JAK inhibitors provides an additional treatment option for this condition with its increasing incidence. Many studies have demonstrated that JAK inhibitors, such as tofacitinib and abrocitinib, play a significant role in the treatment of post-injection granulomas (83–85). Their ability to selectively suppress key inflammatory pathways offers a targeted therapeutic strategy for lesions resistant to conventional treatments.

### 5.5 Other types of cutaneous granulomatous diseases

In addition to the four major granulomatous skin diseases discussed above, JAK inhibitors have also shown promising therapeutic potential in a broader spectrum of granulomatous dermatoses. For instance, granulomatous cheilitis and necrobiotic xanthogranuloma—conditions characterized by persistent granulomatous inflammation and often resistant to conventional therapies—may also benefit from JAK inhibition due to their shared reliance on Th1-mediated immune activation (86–89). Even in rare entities such as granulomatous mycosis fungoides or palisaded neutrophilic and granulomatous dermatitis, early evidence points to the central role of dysregulated cytokine signaling, thereby positioning JAK inhibitors as rational therapeutic options (90–92). These observations collectively highlight the expanding relevance of JAK-STAT pathway inhibition across diverse granulomatous skin disorders, supporting its role as a versatile strategy for conditions with limited treatment alternatives.

## 6 Limitations of the current evidence base

Despite the encouraging therapeutic potential of JAK inhibitors in cutaneous granulomatous diseases, the current evidence base—primarily composed of case reports and small case series—must be interpreted with caution. For instance, open-label studies of tofacitinib in cutaneous sarcoidosis and granuloma annulare have involved only approximately 10 patients, while evidence supporting the use of upadacitinib and abrocitinib similarly derives from sporadic reports of a comparable scale (93–95). Although these preliminary findings are valuable for hypothesis generation and proof-of-concept demonstration, they lack the robustness required to confirm efficacy and safety. A significant gap remains in the form of large-scale, randomized, placebo-controlled trials, which fundamentally limits the clinical persuasiveness of claims regarding the effectiveness of JAK inhibitors. Therefore, it is imperative to conduct well-designed controlled clinical trials to definitively establish efficacy, determine long-term safety, optimize dosing strategies, and identify predictive biomarkers for personalized therapy. Until such high-quality evidence is available, the use of JAK inhibitors in this context should be considered investigational and reserved for carefully selected, treatment-refractory cases. Additionally, treatment responses are heterogeneous, and not all patients achieve satisfactory clinical improvement, potentially due to disease heterogeneity, redundant signaling pathways, or pharmacokinetic variations (96). Relapse following drug discontinuation is another notable challenge, suggesting that JAK inhibition may suppress but not eradicate the underlying inflammatory process. Furthermore, the high cost of these therapies and their off-label use in many granulomatous conditions pose substantial barriers to widespread clinical adoption (97).

## 7 Prospects and outlook

Looking ahead, JAK inhibitors represent a promising frontier in the management of refractory cutaneous granulomatous diseases. The development of topical JAK formulations may offer a targeted approach for localized disease, minimizing systemic exposure and reducing adverse effects (98). Moreover, exploring combination therapies with conventional immunosuppressants or biologic agents may yield synergistic effects, enhancing therapeutic outcomes while allowing dose reduction of individual drugs (99).

The promising yet preliminary nature of the current evidence base underscores an urgent need for high-quality, prospective clinical trials to definitively establish the role of JAK inhibitors in cutaneous granulomatous diseases. Moving beyond anecdotal reports requires meticulously designed studies that can generate robust, generalizable data. A key consideration involves refining patient selection by focusing on those with well-defined, refractory disease and exploring the integration of predictive biomarkers—such as tissue phospho-STAT expression or serum cytokine profiles—to identify subgroups most likely to benefit from treatment. This stratification strategy would enhance trial efficiency and improve the chances of detecting a meaningful clinical signal (100, 101).

The choice of primary endpoints requires careful validation to ensure they capture clinically relevant improvement. While early-phase trials may utilize clinical response rates, definitive Phase III studies should aim to develop and implement a standardized disease activity index that incorporates lesion characteristics, physician assessments, and patient-reported outcomes like quality-of-life measures (102). Furthermore, the field would benefit from adopting innovative trial designs to overcome the challenges of studying rare diseases. A randomized withdrawal design, for instance, is particularly well-suited for demonstrating efficacy maintenance after an initial open-label induction phase. Alternatively, adaptive designs allow for pre-planned modifications based on interim data, making the development process more efficient and flexible.

Finally, establishing a comprehensive safety profile is paramount, given the chronic relapsing nature of these conditions and the known class-level risks of JAK inhibitors. Future trials must incorporate prospective, long-term monitoring protocols for serious infections, major adverse cardiovascular events, thromboembolism, and malignancy. Parallel long-term patient registries will be essential to gather robust real-world safety data that extends beyond the typical duration of controlled clinical trials (103, 104). By systematically addressing these aspects of trial design—patient stratification, endpoint validation, statistical innovation, and safety surveillance—the field can transition from off-label use to evidence-based, personalized therapy for these challenging dermatoses.
